# Cortical modulation through robotic gait training with motor imagery brain-computer interface enhances bladder function in individuals with spinal cord injury

**DOI:** 10.1038/s41598-025-18277-3

**Published:** 2025-10-03

**Authors:** Ericka Raiane da S. Serafini, Cristian D. Guerrero-Mendez, Cristian F. Blanco-Diaz, Fernando da Silva Fiorin, Thayse S. de Albuquerque, André F. O. A. Dantas, Denis Delisle-Rodriguez, Caroline C. do Espírito-Santo

**Affiliations:** 1 Postgraduate Program in Neuroengineering, Edmond and Lily Safra International Institute of Neuroscience, Santos Dumont Institute, Av. Alberto Santos Dumont, 1560-Zona Rural, Macaíba, 59288-899 RN Brazil; 2 Postgraduate Program in Electrical Engineering, Federal University of Espírito Santo (UFES), Av. Fernando Ferrari, 514, Vitória, 29075-910 ES Brazil

**Keywords:** Spinal cord injury, Neurogenic bladder, Robotic gait training, Motor imagery, Brain computer interface, Neuroscience, Engineering

## Abstract

Neurogenic bladder (NB) dysfunction in individuals with complete spinal cord injury (SCI) is a condition that significantly affects quality of life. Despite the prevalence of interventions, there is a substantial gap in effective treatments for this dysfunction. This study proposes robotic-assisted gait training combined with motor imagery (MI)-based brain-computer interface (BCI) to induce improved cortical modulation, and consequently improve bladder function in patients with SCI. The study involved seven men with complete and chronic SCI in a protocol comprising 24 sessions of robotic-assisted walking with BCI and MI. This regimen was designed to teach both mu (*µ*, 8–12 Hz) and beta (*β*, 15–20 Hz) modulation through MI practices using multi-channel EEG neurofeedback (NFB), focusing on sensorimotor rhythm (SMR) activation. Clinical outcomes were measured using the neurogenic bladder symptom score (NBSS), which revealed substantial improvements in bladder control among participants. EEG analysis confirmed a significant correlation between modulation of *µ* and *β* rhythms with decreased NBSS scores. Our findings support that robotic-assisted gait training combined with MI-based BCI effectively modulates with more precision the cortical *µ* and *β* rhythms and improves NB dysfunction in SCI individuals.

**Supplementary Information:**

The online version contains supplementary material available at 10.1038/s41598-025-18277-3.

## Introduction

Spinal cord injury (SCI) affects the quality of life by damaging the central and peripheral nervous systems (CNS/PNS)^1,2^. Among several comorbidities, neurogenic bladder (NB) remains intractable^3^leading to the need to develop new approaches to treatment and symptom reduction^4^. NB is a component of neurogenic lower urinary tract dysfunction (NLUTD), which is characterized by the inability to control urine elimination. SCI individuals require intermittent catheterization, which has been associated with infection, injury to organs of the urinary tract, and an increased risk of developing bladder cancer^5–8^.

SCI-induced NLUTD involves changes in neural control and bladder function, including alterations in the neuronal circuit between the brain and the pontine micturition center, which is responsible for managing voiding^9^. Additionally, the autonomic nervous system (ANS), and pelvic floor muscles, which help control urination and support the bladder, are impaired after SCI^10,11^.

Trunk control mediated by muscles relies on somatosensory system inputs to generate coordinated movement patterns^11,12^. Thus, the ANS, CNS, and PNS are associated with the loss of the sensorimotor system, maladaptive cortical reorganizations, and NB after SCI^13–16^. Interestingly, tomographic images have revealed the existence of an extensive brain network that includes the frontal lobe, which is crucial for the modulation of micturition and urine storage^17–20^.

Electroencephalography (EEG) recordings have shown modulation of high-beta oscillations through robotic-assisted gait training and stimulation, improving gait recovery in SCI individuals^14,21,22^. In this context, the Lokomat, a robotic device designed to assist gait after SCI, can activate damaged sensorimotor rhythms (SMRs), such as mu (*µ*, 8–12 Hz) and beta (*β*, 13–30 Hz)^23,24^. Recently, our group showed that combining motor imagery (MI), visual neurofeedback (NFB), and Lokomat improves sensory and autonomic outcomes after SCI^25^. Additionally, NFB and brain-computer interfaces (BCI) using movement attempts^26^as well as different isolated rehabilitation interventions^27^also induces activation of SMRs, playing a crucial role in physical restoration^26,27^.

Although there is evidence of an improvement in sensorimotor symptoms through these techniques, treatments for SCI-induced NB remain inconclusive. Therefore, it is important to investigate whether improving the modulation of cortical motor rhythms through MI-based BCI training with the Lokomat can improve bladder function in individuals with SCI. The overall goal of this study is to verify the impact of *µ* and *β* modulation through MI-based BCI with multi-channel EEG NFB linked to robotic-assisted gait training on NB in individuals with complete SCI.

## Methods

### Ethical approval and participants

This project was approved by the Research Ethics Committee of the Santos Dumont Institute (CAAE: 53127921.2.0000.0129), and conducted in accordance with the Helsinki Declaration of 1975, revised in 2013.

This exploratory study of type case series was carried out with seven male individuals. Participants were selected for convenience, with ages between 15 and 60 years. The inclusion criteria were individuals clinically diagnosed with complete and chronic motor SCI (more than 1 year after injury), classified as A or B on the American Spinal Injury Association (ASIA) impairment scale^28^. Furthermore, their degree of spasticity in lower limbs should be less than 2 considering Modified Ashworth Scale (MAS) perform kinesthetic MI assessed by the kinesthetic and visual imagery questionnaire-10 (KVIQ-10)^29^. Exclusion criteria were the presence of symptoms because of dysautonomia, orthostatic hypotension, osteoporosis, skin lesions, or contractures (Table 1). All participants were diagnosed with NB by urodynamic examination within the first year of injury.

**Table 1:** Sample characterization before intervention.

Subject	Age (years)	Neurological level and severity	Injury time (years)	Actual Management	KVIQ-10
S1	34	T6, ASIA B	3	Intermittent self-catheterization	19
S2	42	T5, ASIA A	2	Intermittent self-catheterization	18
S3	50	T9, ASIA A	6	Intermittent self-catheterization	13
S4	37	T4, ASIA A	5	Intermittent self-catheterization	7
S5	58	T9, ASIA A	2	Intermittent self-catheterization	11
S6	15	T4, ASIA A	1	Intermittent self-catheterization	16
S7	36	T4, ASIA A	6	Intermittent self-catheterization	11

### Clinical evaluation

Firstly, we used the ASIA, KVIQ-10 and MAS clinical tools to sample characterization.

#### American spinal injury association (ASIA) impairment scale

The ASIA scale was used to categorize the level and severity of SCI. This scale classifies patients into A to E, where A represents a complete absence of motor and sensory function in the sacral segments S4-S5, and E indicates normal motor and sensory function. The ASIA Scale is employed for a detailed evaluation of the impact of SCI, classifying the injury as complete or incomplete. It involves the analysis of the neurological level, the assessment of muscle strength, and sensitivity to stimuli such as light touch and tactile discrimination. Low scores indicate a greater likelihood of recovery^30^. To diagnose the completeness of SCI, the S4-S5 questionnaire was used to assess sensation and motor control in the perineal region and determine the integrity of S4-S5 segments of the spinal cord. Sensory assessment assesses light touch and pinprick sensation in dermatomes S4-S5, scored on a 3-scores scale: 0 for absent, 1 for impaired and 2 for normal. The motor assessment assesses voluntary anal contraction, scored as 0 for absent or 1 for present^31^.

#### Kinesthetic and visual imagery questionnaire-10 (KVIQ- 10)

The KVIQ-10 questionnaire is an assessment tool that measures an individual’s capacity for visualization and mental imagery. It evaluates the individual’s mental imagery ability both visually and kinesthetically. It assesses 5 items, with each item rated on a scale from 1 to 5, where 1 indicates a very weak or absent mental image and 5 indicates a detailed mental image^29^.

#### Modified Ashworth scale (MAS)

The MAS is a clinical assessment tool used to measure the degree of muscle spasticity in patients with neurological conditions. It quantifies the resistance to passive movement in various joints, assigning scores from 0 to 4, where 0 indicates no spasticity and 4 indicates severe spasticity^32,33^. The Neurogenic Bladder Symptom Score (NBSS) modified functional reach test (mFRT) and the ASIA sensory score in the trunk were used to assess the impact of the proposed intervention using multi-channel EEG NFB based BCI.

#### Neurogenic bladder symptom score (NBSS)

The NBSS is a tool to quantify bladder-related symptoms in individuals with neurogenic urinary tract dysfunction, including SCI. The NBSS consists of 24 items assessing bladder symptoms in three different do- mains: incontinence (scored from 0 to 29), storage and micturition (scored from 0 to 22), and consequences (scored from 0 to 23), with scores being higher the greater the neurogenic bladder-related symptoms. In addition, there is a single global question regarding urinary quality of life, scored from 0 (satisfied) to 4 (dissatisfied). Higher scores indicate greater impairment^34^. The minimal clinically important difference (MCID) for the NBSS in individuals with incomplete SCI was reported to be at least 5 scores^35^.

#### Modified functional reach test (mFRT)

The mFRT was used in sitting position to measure the maximum distance that the participant reached targets forward, a right side, or left side, and return to the starting position independently. In the study, volunteers took the measurement from the distal tip of the third metacarpal along a ruler fixed on the wall and positioned at the height of the acromion of the participant’s dominant arm. To obtain the score, the average on three repetitions was calculated^36,37^.

#### ASIA sensory score in the trunk

To assess trunk sensitivity, we used the ASIA light touch and pin prick methods on the right and left sides of the participants’ trunks. We then summed the responses obtained for each type of stimulus on both sides, calculating a total score^30^.

### Proposed intervention

**Fig. 1:**
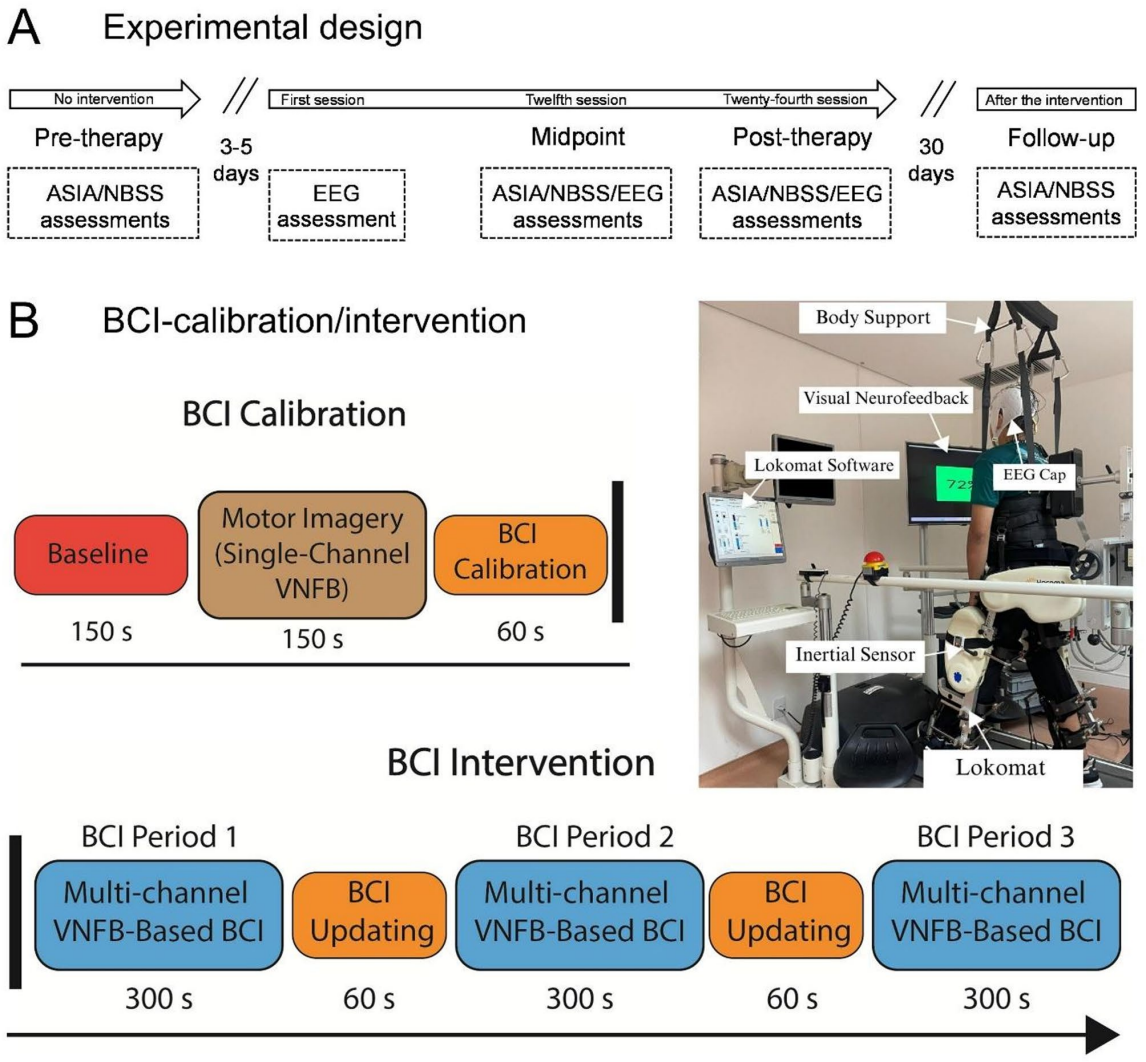
(**A**) Experimental design showing a detailed timeline of assessments and treatments. (**B**) Block diagram illustrating the proposed BCI for intervention with Lokomat-assisted gait training and NFB presentation.

The clinical intervention was conducted using a multi-channel EEG NFB- based BCI system, allowing SCI individuals to practice MI by learning to modulate both *µ* and *β* rhythms during robotic-assisted gait training with the Lokomat^®^ (Hocoma AG, Switzerland). Before the intervention (pre-therapy assessment), participants were evaluated using the ASIA sensory score for the trunk, the NBSS for neurogenic bladder symptoms, and the mFRT to assess trunk balance. After 3–5 days, participants began the intervention using the complete BCI system while performing gait MI during Lokomat training. Each participant completed 24 sessions over 12 weeks (two sessions per week). To better understand the impact of the intervention, each participant was re-assessed after completing the 12th session (midpoint assessment), 24th session (post-therapy assessment) and on the 30th day after finishing the intervention (follow-up). No intervention was administered in the follow-up period (Fig. 1A).

Each 43-min session was structured into approximately 20 min for preparation and 23 min for the BCI intervention. During the preparation phase, the participant, seated in their wheelchair, donned an EEG cap and briefly practiced kinesthetic MI of walking. Afterwards, they were positioned in the Lokomat to begin the main training phase with the EEG-based BCI system. The training began with a calibration stage lasting 6 min, divided into two 150-s periods: the first involved passive robotic gait without MI (baseline), and the second involved gait MI during passive walking, accompanied by single-channel NFB provided from the Cz electrode. The reason for initially using a single EEG channel (Cz) during the first calibration is to simplify the task for users at the early stage, providing single-channel NFB focused on a brain region most associated with lower-limb MI. It is worth noting that this step does not use a classification system to better guide the individual, but it uses EEG data from the first 150 s as a Baseline (or reference), for gait imagery training with single-channel NFB. Therefore, this phase allows the user to begin learning the self-modulation of *µ* and *β* rhythms. As a result, more reliable MI EEG data together with baseline data are collected for the BCI calibration.

In addition to the single-channel NFB, our BCI system also provides multichannel EEG neurofeedback, as explained in Blanco-Diaz et al., (2024)^38^. For this purpose, multi-channel EEG features using Riemannian covariance matrices are employed to accurately classify both walking without MI and walking with MI by using Linear Discriminant Analysis (LDA). The classification output is used in the visual neurofeedback, which uses a NFB model obtained from multiple electrodes over the foot area (FC1, FC2, CP1, CP2, C1, C2, and Cz). The NFB model is computed through a cluster analysis based on Euclidean distances taking as a reference the weighted average feature vector (that corresponds to MI features) based on both *µ* and *β* rhythm oscillations. During the BCI recalibration, the weighted average feature vector is updated, using only features classified as gait MI in each period of 5 min (Fig. 1B). Following this, participants engaged in 17 min of active BCI training using multi-channel EEG-based NFB during Lokomat-assisted gait training. During this stage, participants practiced gait MI while receiving visual feedback derived from the real-time modulation of *µ* and *β* rhythms, walking passively in the Lokomat.

The visual neurofeedback percentage is calculated in two-ways: (1) computing the brain wave power changes with respect to the Baseline using 1s-EEG epochs; and (2) computing the Euclidean distance between a new feature vector classified as MI and the weighted average feature vector corresponding to the MI class. The brain wave power change in percentage is computed based on the relative power change in *µ* and *β* bands over sensorimotor areas, using Eqs. (6–7) in Blanco-Diaz et al., (2024)^38^ and a threshold value at 40% to deliver two visual feedback, green for relative power change values above 40%, and red otherwise. The second Neurofeedback model uses the relative power change with respect to the baseline from the training EEG data, in order to weight Riemannian covariance features corresponding to the MI class, and consequently obtain its weighted mean feature vector. Then, Euclidean distances between these feature vectors and its weighted mean feature vector are computed, and interpolated into percentage scores, given the highest scores (near 100%) to distances close to 0, and lowest scores (near 40%) otherwise. Notice that the highest score means that a new feature is nearest to the expected values, and it is considered as a strong *µ* and *β* modulation and appropriate MI execution.

The therapist guided the participant throughout the session using standardized verbal commands to promote consistent MI engagement, which are presented as follows.

- During the Initial 150 s (baseline): *“You Will Walk With the assistance of Lokomat and continue to look at this screen without performing MI.”*
- From 150 s to 300 s (single-channel EEG NFB): *“You will imagine yourself walking on this treadmill with the assistance of the Lokomat*,* imagining that you are walking by placing one foot in front of the other.”*
- Reinforcement occurred every 90 s, except during rest intervals, the participant passively walked uninterrupted throughout all time in Lokomat with 70% body weight support and 100% guidance force at a speed of 1 km/h. These Lokomat parameters remained unchanged throughout the intervention.

### EEG data acquisition to Multi-channel EEG NFB based BCI

Initially, the scalps of the individuals were cleaned using 70% alcohol and an electroconductive gel (EasyCAP) was applied to ensure that the impedance of the EEG channels was kept below 10 kΩ. EEG data were collected using the V-Amp 16 amplifier (Brain Products) and sixteen actiCAP Xpress dry electrodes located on FP1, FP2, F3, F4, FC1, FC2, C1, C2, C3, C4, Cz, CP2, CP1, P4, Pz and P3, according to the international 10–10 system for scalp mapping. Data acquisition is synchronized with the OpenVibe Acquisition Software for real-time processing, and recording data was set at a sampling rate of 512 Hz.

### EEG data processing and analysis

EEG signals were band-pass filtered using a fourth-order zero phase Butterworth filter in the *µ* and *β* frequency bands^39,40^. A common average reference filter was also employed to remove common interference between EEG channels. The signals were segmented according to the gait cycle using the JAMA accelerometer^41^positioned on the participant’s left leg near the knee joint to accurately identify the gait cycles. The relative power spectral density (PSD) was then calculated on the EEG segments for each frequency band, and the mean PSD values were then computed for both the baseline and gait MI conditions. All data were processed using MATLAB (version R2021a, MathWorks Inc.).

### Statistical analysis

The Shapiro-Wilk test was used to assess the distribution of the data. The Friedman test was used for each domain of the NBSS scale and mFRT, followed by a post hoc analysis using the Conover’s test with Bonferroni correction to adjust for multiple comparisons (for non-parametric data). Furthermore, spearman correlation analysis was conducted to evaluate the relationship between the domains of the NBSS scale and the ASIA sensory score in the trunk, the mRFT, and EEG power data. The Spearman correlation coefficient classification is as follows: Moderate (0.40 to 0.59 or − 0.40 to − 0.59); Strong (0.60 to 0.79 or − 0.60 to − 0.79); Very Strong (0.80 to 1.00 or − 0.80 to − 1.00). A *p*-value < 0.05 was considered statistically significant for the Friedman test and correlations.

## Results

### Effects of multi-channel EEG NFB based BCI combined with Lokomat on the NBSS scores

**Fig. 2:**
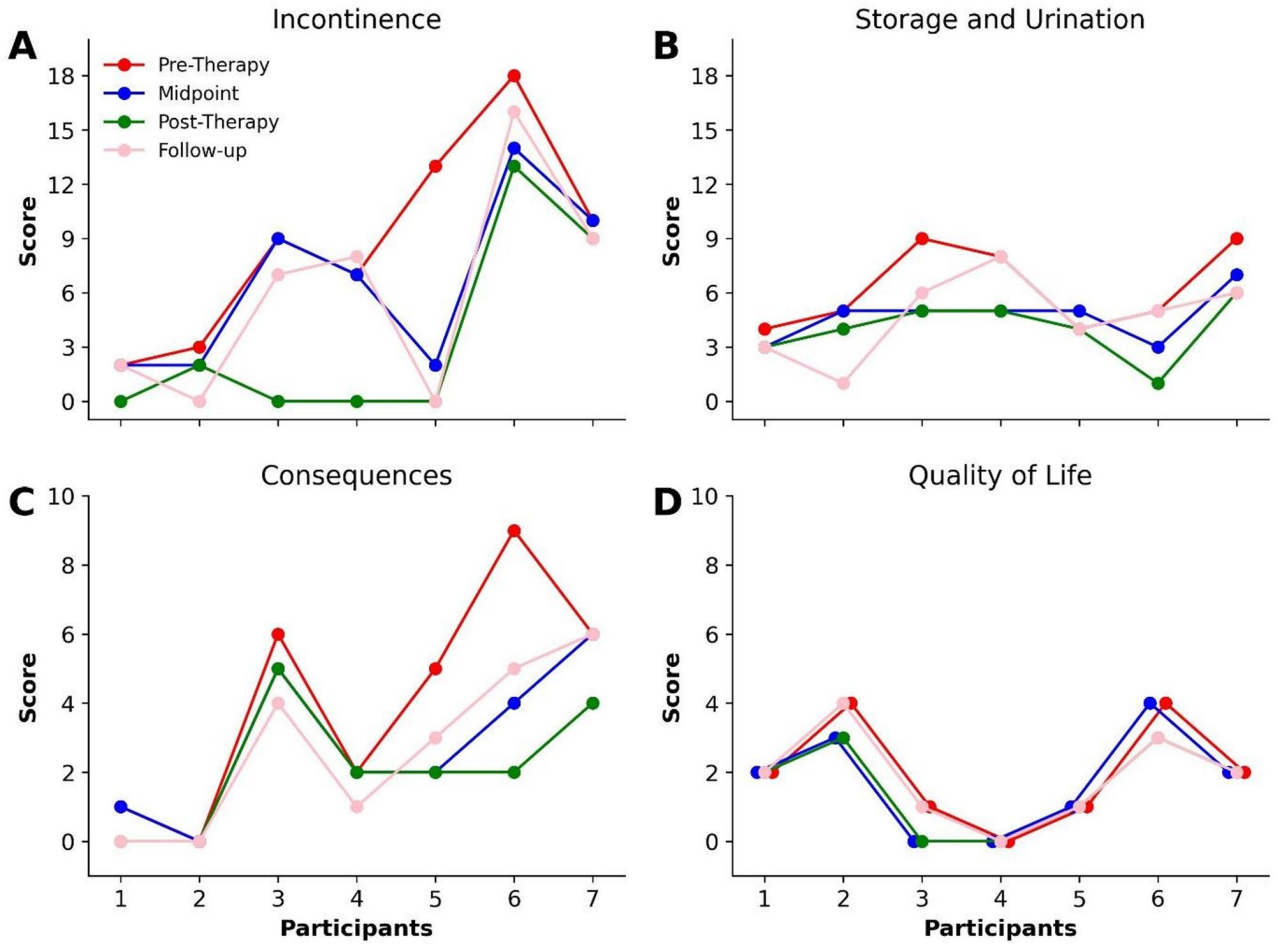
Progression of NBSS scale domain scores carried out in four clinical assessments on the seven participants with SCI, which were treated with robotic-assisted gait training with MI-based BCI. (**A**) Incontinence score; (**B**) Storage and urination score; (**C**) Consequences score; (**D**) Quality of life score.

Figure 2 shows the individual NBSS scores. Subject 1 exhibited a notable improvement in the incontinence, decreasing from 2 to 0 (pre-therapy to post-therapy), which indicates the absence of incontinence (Subject 1; Fig. 2A). However, in the follow-up evaluation, his scores returned to pre-therapy score (Subject 1; Fig. 2A). Furthermore, decreased by 1 score in the storage and urination, and consequences categories, maintaining this outcome until follow-up evaluation (Subject 1; Fig. 2C and B, and 2D). Subject 2 presented a significant improvement by reducing 1 score in incontinence, 2 scores in storage and urination, and 1 score in the quality-of-life category (Subject 2; Fig. 2A, B and D) when comparing pre-therapy with post- therapy. Interestingly, in the follow-up evaluation, there is a reduction of 2 and 3 scores for incontinence and storage and urination, respectively, while quality of life increased 1 score, compared to pre-therapy (Subject 2; Fig. 2A, B and D).

Subject 3 had a 9-scores improvement in the incontinence, a 4-scores improvement in storage and urination, a 1-score reduction in consequences, as well as a 1-score improvement in the quality-of-life domain when comparing pre-therapy to post-therapy (Subject 3; Fig. 2A, B, C and D). Furthermore, subject 3 also achieved the MCID suggesting a considerable improvement of 15 scores. However, in the follow-up evaluation, the incontinence scores returned almost to the pre-therapy stage, while storage and urination and quality of life returned 1 score from post-therapy values (Subject 3; Fig. 2A, B and D). In contrast, the consequence category had a decrease of 1 score from post-therapy to follow-up (Subject 3; Fig. 2C).

Subject 4 decreased from 7 scores in the urinary incontinence, achieving urinary continence, and decreased 3 scores in storage and urination when comparing pre-therapy vs. post-therapy (Subject 4; Fig. 2A and B), with a reduction of 10 scores in the total NBSS score. In the follow-up analysis, the scores for incontinence and storage and urination return to pre-therapy values, with an impairment of 1 score for incontinence (Subject 4; Fig. 2A and B). However, there is an improvement in the consequences category of 1 score in the follow-up analysis compared to post-therapy (Subject 4; Fig. 2C). Thus, subject 4 also achieved the MCID change in post-therapy. Furthermore, a transition from intermittent catheterization to spontaneous urination was reported.

Subject 5 reduced 13 scores in incontinence and 3 scores in the consequences categories from pre-therapy to post-therapy (Subject 5; Fig. 2A and C), reaching the MCID change of at least 5 scores. Furthermore, in the incontinence follow-up analysis, the post-therapy score was maintained, while for consequences there was an increased 1 score compared to the post-therapy analysis (Subject 5; Fig. 2A and C). However, no changes were observed in the storage and urination domain, as well as in the quality of life (Subject 5; Fig. 2B and D).

Subject 6 decreased 5 scores in incontinence, 4 scores in storage and urination, 7 scores in consequences, and 1 score in quality of life, all comparing pre-therapy vs. post-therapy (Subject 6; Fig. 2A, B, C and D), resulting in a reduction of 17 scores on the NBSS scale. Finally, Subject 7 reduced 1 score in incontinence, 3 in storage and urination, and 2 scores in consequences when comparison is between pre-therapy vs. post-therapy (Subject 7; Fig. 2A, B and C), resulting in a reduction of 6 scores in NB symptoms assessed by NBSS. In the follow-up analysis, there was a maintenance in incontinence and storage and urination scores (Subject 7; Fig. 2A and B), while the consequences values returned to the pre-therapy scores (Subject 7; Fig. 2C). In general, the interventions had a positive impact on NB symptoms in SCI subjects, showing that six of seven subjects achieved a clinically important improvement on the NBSS scale.

**Fig. 3:**
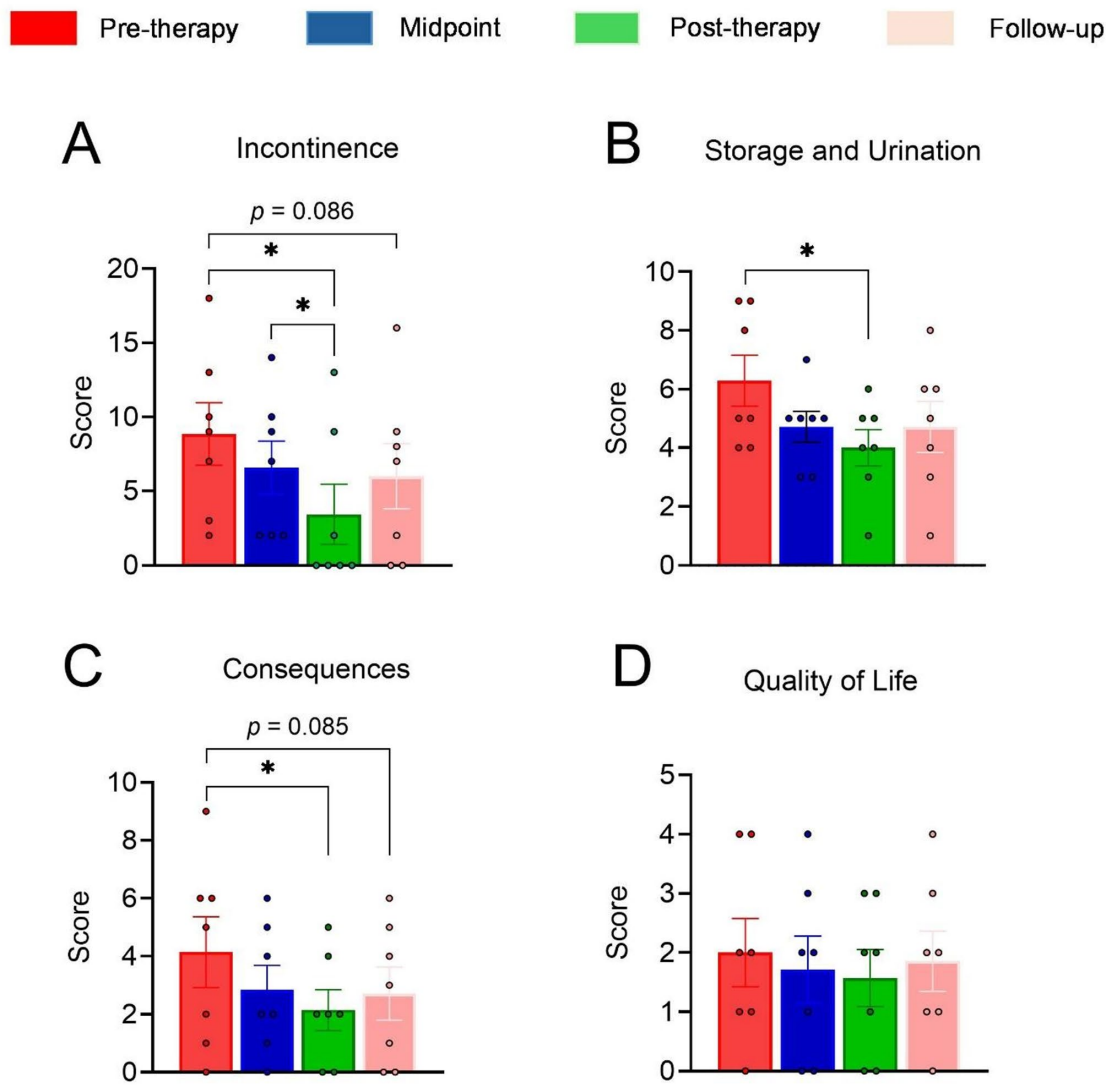
Clinical effects applying the proposed treatment. (**A**) Incontinence score; (**B**) Storage and Urination score; (**C**) Consequences score; (**D**) Quality of Life score. *n* = 7 individuals. Data analyses were conducted using Friedman non-parametric tests. Data are expressed as the mean ± SEM. Post hoc analyses were performed using Conover’s test with Bonferroni correction when appropriate. *p < 0.05.

Analysis of multiple measurements using the Friedman test showed a significant difference between days of evaluations for the incontinence symptom (F [3, 24] = 12.150; *p* = 0.007; Fig. 3A). Post hoc analysis revealed that there was a significant decrease in the score post-therapy evaluation when compared with the pre-therapy (*p* = 0.01; Fig. 3A).

Furthermore, the incontinence score had a more significant decrease in the post-therapy evaluation compared to the midpoint evaluation (*p* = 0.021; Fig. 3A). Although the difference between pre-therapy and follow-up did not reach statistical significance (*p* = 0.086), the results showed a trend toward improvement, suggesting a potential effect of the maintenance protocol in the incontinence domain when evaluated 30 days after the end of treatment. Statistical analysis of storage and urination revealed a significant decrease throughout the assessment days (F[3, 24] = 9.214; *p* = 0.027; Fig. 3B). The post hoc test demonstrated significant differences between pre-therapy and post-therapy evaluations (*p* = 0.009; Fig. 3B).

The consequences parameters also had a significant change after treatment (F[3, 24] = 8.625; *p* = 0.035; Fig. 3C). Post hoc analysis shows a statistically significant reduction in post-therapy evaluation compared with the pre-therapy evaluation (*p* = 0.023; Fig. 3C). A non-statistical reduction was observed in follow-up compared to the pre-therapy (*p* = 0.085). No statistical differences were observed for quality of life throughout the assessment days (F[3, 24] = 5.000; *p* = 0.172; Fig. 3D).

### Effects of multi-channel EEG NFB based BCI combined with Lokomat on ASIA score

Although statistical analysis did not show significant differences between the evaluations (F[3, 24] = 2.1320; *p* = 0.1318), six out of seven individuals presented an increase in trunk sensory score post-therapy compared to the pre-therapy (see Table 1 of the Supplementary Materials), indicating clinical improvement.

**Fig. 4:**
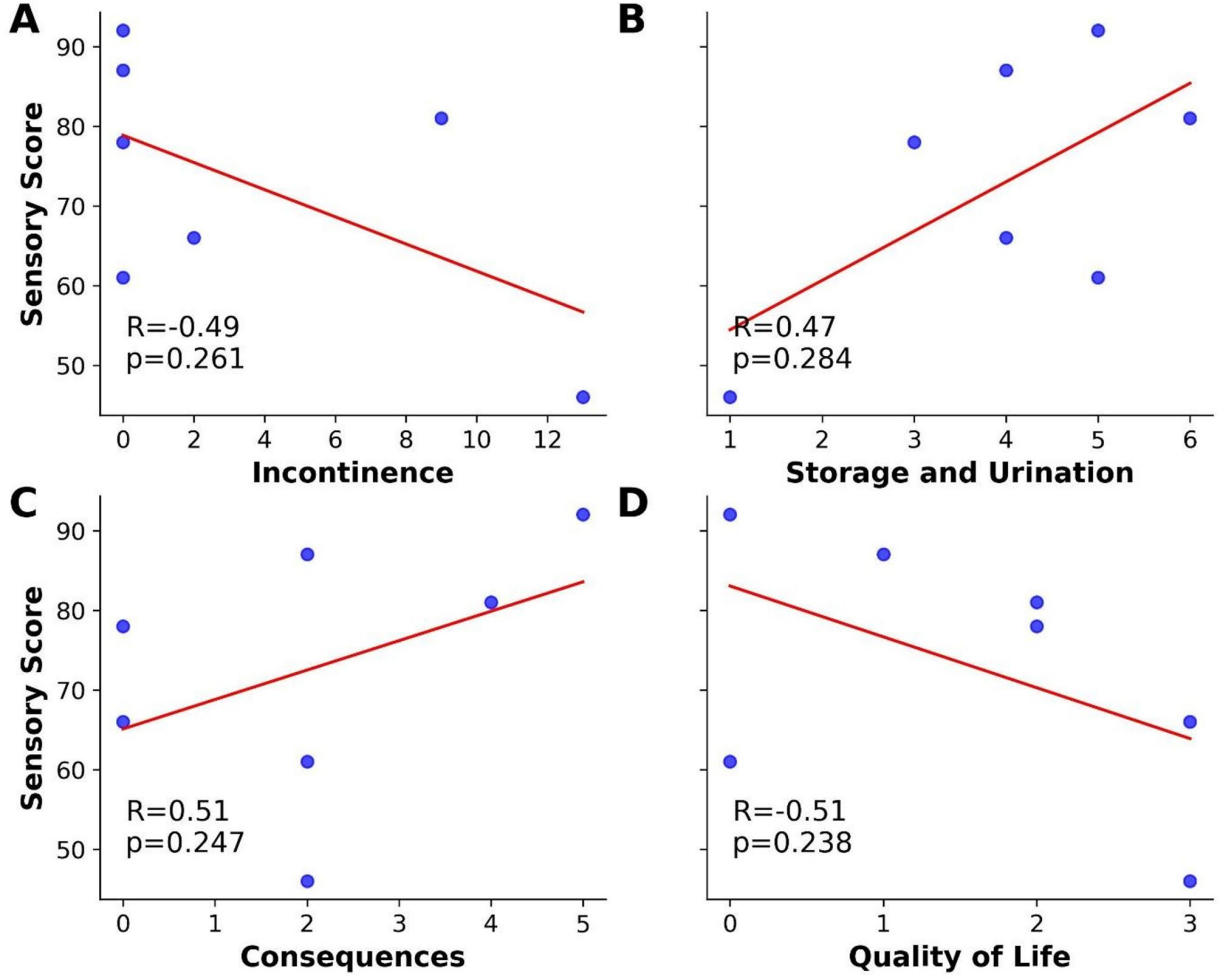
Correlation between ASIA sensory score on the trunk and NBSS domains in seven SCI individuals post-treated with robotic-assisted gait training with multi-channel EEG NFB based BCI. The y-axis shows the ASIA sensory score in the trunk and x-axis the domains (**A**) incontinence, (**B**) storage and urination, (**C**) consequences, and (**D**) quality of life. Measurements in each individual are represented by the blue dots. *n* = 7 individuals. The red line represents the trend line positive and/or negative.

Spearman correlation was performed between the ASIA sensory score in the trunk and the NBSS domain post-therapy. The results observed showed no significant correlations for all domains with the sensory score (Fig. 4).

### Multi-channel EEG NFB based BCI combined with Lokomat on mFRT

The scores obtained in the mFRT evaluation are presented in Table 2 of the Supplementary Materials, including both frontal and lateral assessments across all evaluation points of the experimental design.

**Fig. 5:**
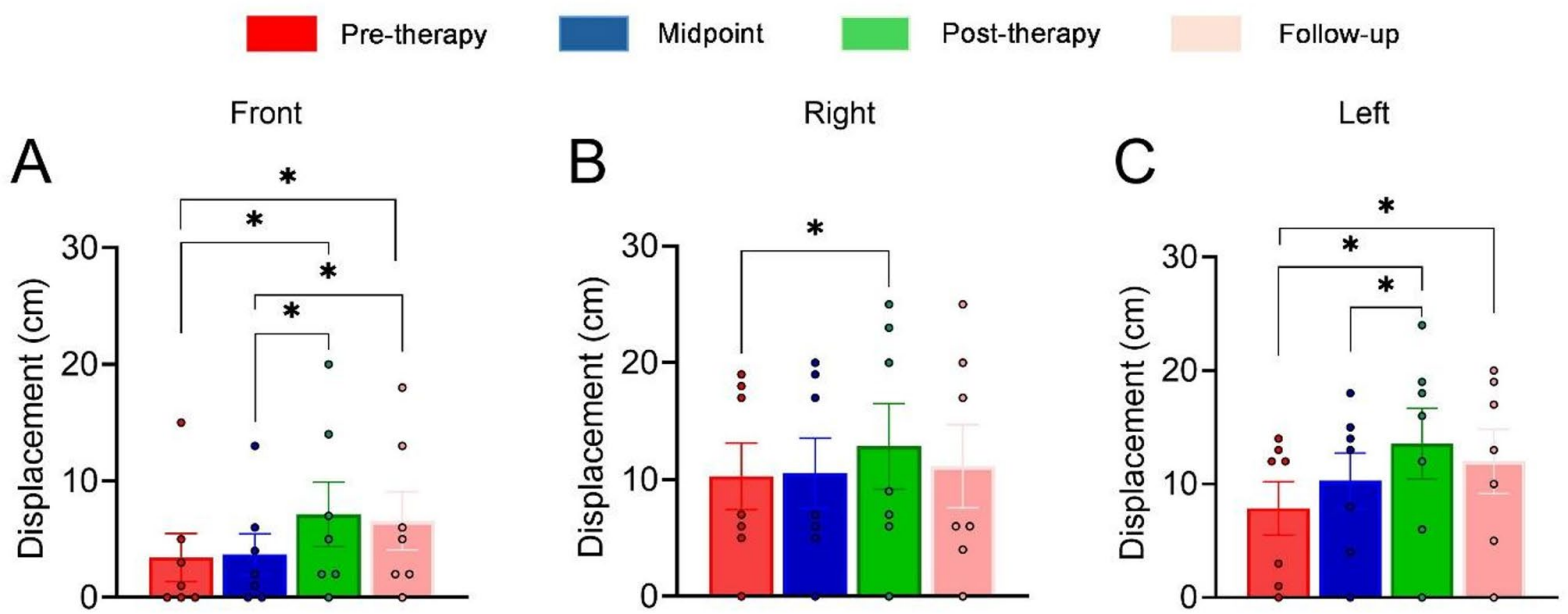
Effects of treatment considering different evaluation days using the mFRT. (**A**) Frontal; (**B**) Right; (**C**) Left. Data analyses were conducted using the Friedman non-parametric test, and the results are expressed as the mean ± SEM. Post hoc analyses were performed using the Conover’s test with Bonferroni correction. * *p* < 0.05.

Statistical analysis showed significant differences in frontal (F [3, 24] = 15.0000; *p* = 0.002; Fig. 5A), right (F[3, 24] = 8.647; *p* = 0.034; Fig. 5B), and left (F[3, 24] = 13.475, *p* = 0.004; Fig. 5C) displacement evaluations in centimeters. Post hoc analysis showed significant differences between the pre-therapy and post-therapy (*p* < 0.001; Fig. 5A) and pre-therapy compared with follow-up (*p* = 0.001; Fig. 5A) to frontal evaluations. Moreover, there was an improvement in the post-therapy compared to the midpoint (*p* = 0.001; Fig. 5A) and follow-up compared with midpoint (*p* = 0.02; Fig. 5A). To the right evaluation, there was a significant increase in the post-therapy compared to the pre-therapy (*p* = 0.021; Fig. 5B). In the left, post hoc analysis showed significant differences in pre-therapy and post-therapy (*p* < 0.001; Fig. 5C), and in pre-therapy and follow-up (*p* = 0.05; Fig. 5C). Moreover, significant differences were observed between midpoint and post-therapy evaluations (*p* = 0.005; Fig. 5C).

**Fig. 6:**
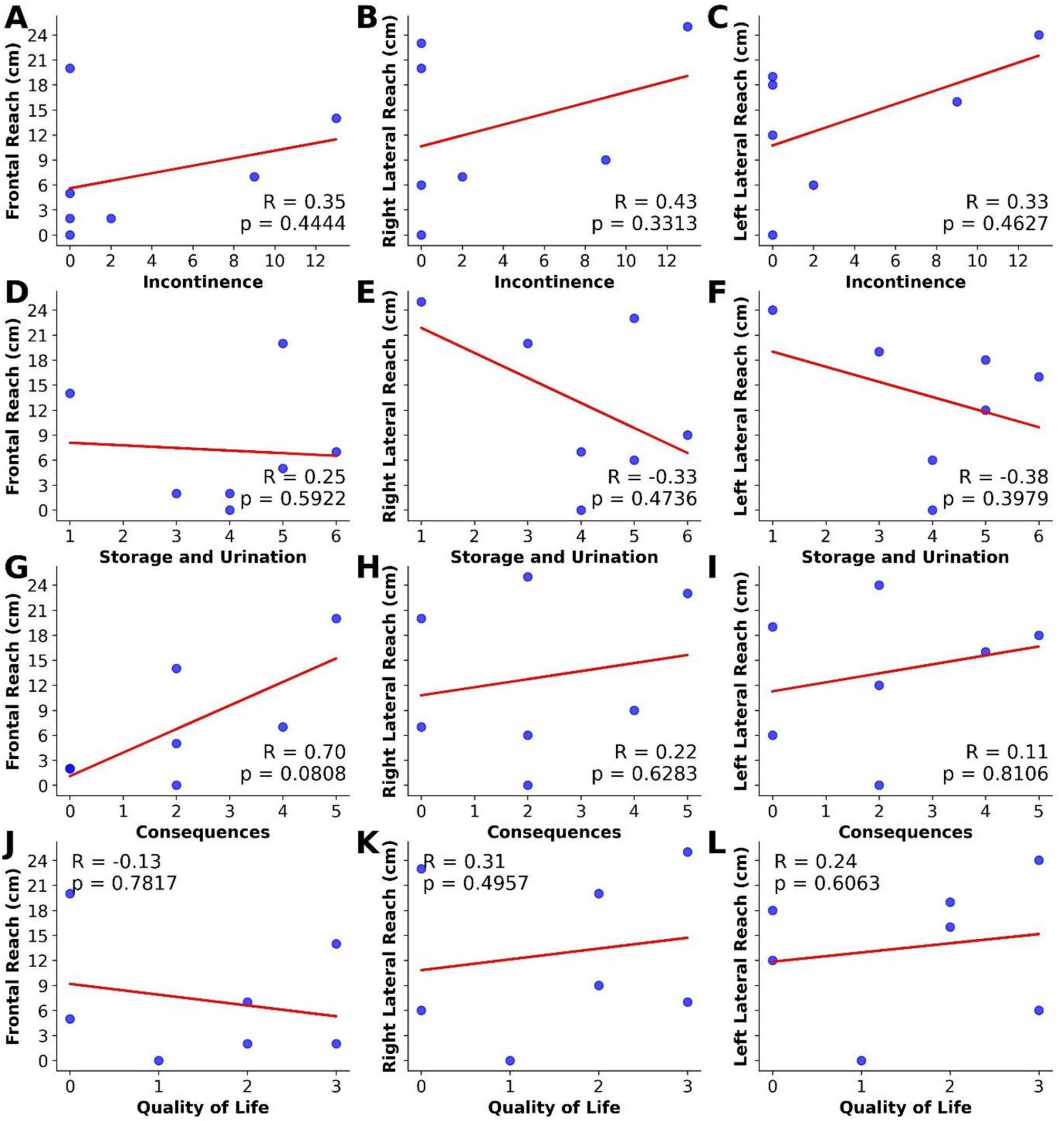
Correlation between functional range test and NBSS domains post-therapy of seven SCI individuals treated with robotic-assisted gait training with multi-channel EEG NFB based BCI. The y-axis shows the mFRT scores and the x-axis the NBSS scores. (**A**) Front side vs. incontinence; (**B**) Right side vs. incontinence; (**C**) Left side vs. incontinence; (**D**) Frontal side vs. storage and urination; (**E**) Right side vs. storage and urination; (**F**) Left side vs. storage and urination; (**G**) Frontal side vs. consequences; (**H**) Right side vs. consequences; (**I**) Left side vs. consequences; (**J**) Frontal side vs. quality of life; (**K**) Right side vs. quality of life; and (**L**) Left side vs. quality of life. Measurements in each individual are represented by the blue dots. *n* = 7 individuals. The red line represents the trend line positive and/or negative.

Regarding the statistical differences between the pre-therapy and post-therapy assessments, we tested if correlation between mFRT and NBSS was significant. However, no significant correlation was observed in this analysis (Fig. 6).

### Correlation of the NBSS scores with the *µ* and *β* SMRs

**Fig. 7:**
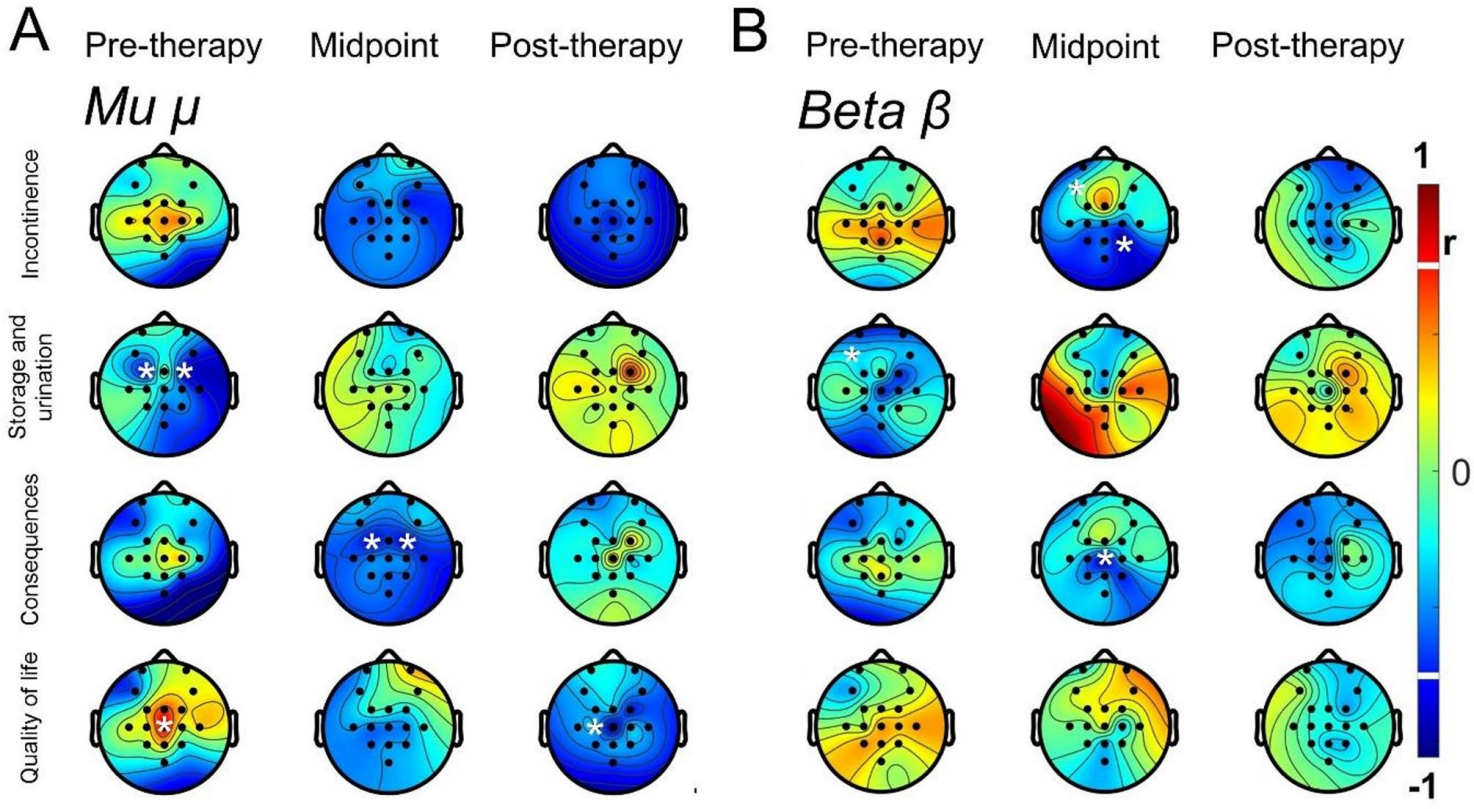
Topographic maps visualizing the correlation between motor rhythms and NBSS scores at the pre-therapy, midpoint, and post-therapy evaluations. Rhythms correspond to power changes, which were correlated with scores in incontinence, storage and urination, consequences, and quality of life. * indicate correlations with *p* < 0.05 in the channel representing the brain area, when the correlation value falls below or exceeds the thresholds marked by the white lines on the color bar.

Statistical analysis showed a significant negative correlation in the frontal-central region (FC1/FC2 channel) to *µ* rhythm and consequence scores in the midpoint evaluation (FC1 channel, *r* = − 0.7928; *p* = 0.0397; Fig. 7A; FC2 channel, *r* = − 0.8108; *p* = 0.0349; Fig. 7A). Furthermore, a negative correlation was observed between the rhythm of *µ* in the somatosensory cortex and the quality of life in the post-therapy (C1 channel, *r* = − 0.8994; *p* = 0.0127; Fig. 7A).

Although no significant correlation was observed for the rhythm *µ* in the motor cortex, there was a tendency for negative correlation in the post-therapy evaluation of the incontinence domain (Cz channel, *r* = − 0.7587; *p* = 0.0762; Fig. 7A). Interestingly, positive and negative correlations were observed in the pre-therapy evaluation, before any intervention. Data showed negative correlation between *µ* rhythm in the frontal-central region with storage and urination domain (FC1 channel, *r* = − 0.7709; *p* = 0.0508; Fig. 7A; FC2 channel, *r* = − 0.7893; *p* = 0.0413; Fig. 7A), as well as positive correlation between *µ* rhythm in the motor cortex and quality of life in the pre-therapy evaluation (Cz channel, *r* = 0.826; *p* = 0.0381; Fig. 7A). Interestingly, all these statistical correlations were not significant in midpoint, implicating in early maladaptive correlations related to NB symptoms depending on brain areas evaluated.

Regarding *β* rhythm, statistical analysis showed a significant negative correlation in the frontal lobe and parietal central areas for the incontinence domain in midpoint evaluation (FP1 channel, *r* = − 0.8154; *p* = 0.0381; Fig. 7B; CP2 channel, *r* = − 0.9266; *p* = 0.0095; Fig. 7B). Moreover, significant negative correlation was observed between *β* rhythm and the domain of consequences in the midpoint evaluation of the motor cortex (Cz channel, *r* = − 0.8829; *p* = 0.0135; Fig. 7B). In the pre-therapy evaluation, there was also a significant negative correlation for rhythm *β* in the frontal lobe and the storage and urination domain (FP1 channel, *r* = − 0.876; *p* = 0.0354; Fig. 7B).

## Discussion

The present study demonstrated the impact of the interventions on NB symptoms in subjects with SCI, showing that six out of seven subjects achieved a clinically important improvement on the NBSS scale. In addition, the present protocol demonstrated efficiency in improving the mFRT, while it did not present beneficial results in trunk sensibility. Furthermore, PSDs of rhythms *µ* and *β* suggest substantial cortical reorganization in response to the combined intervention, which are associated with an improvement in NB symptoms after SCI.

Although single-modality interventions such as exoskeleton-based gait training show promise, their isolated effects on NLUTD remain inconclusive. For example, Williams et al.^11^ reported that active gait training with the Ekso exoskeleton led to greater pelvic floor muscle recruitment compared to passive Lokomat training, but the improvements in NB symptoms, after in both modality of training, were not consistent across participants. Furthermore, the study cited above assessed bladder function using urodynamics and the Qualiveen-30, which limits direct comparisons with our findings. In contrast, multimodal interventions, especially those integrating active gait training with or without BCI, have shown more robust effects. Shokur et al.^42^ demonstrated that after 28 months of mixed rehabilitation program, individuals with complete SCI exhibited improved awareness of bladder fullness and enhanced voluntary control during catheterization in more than half of the participants. Despite the limitations in standardized assessment tools to measure NB symptoms, such as International Lower Urinary Tract Function Basic Data Set^43^it is evident that active gait training contributes positively to bladder function recovery and that cortical involvement is a key mechanism underlying this improvement.

In this context, our motivation to explore the role cortical activity on NLUTD emerge from the findings of Serafini et al. (2024)^25^who demonstrated the self-modulation of *µ* and *β* rhythms on Cz area (representing motor primary cortex) via single-visual NFB in concomitance with Lokomat training improved NBSS, an outcome measure validity to SCI population^34^.

The new proposal of the present study is to use a novel MI-based BCI that provides multichannel EEG visual neurofeedback while an individual receives gait training in Lokomat. However, this system does not depend on external signals outside the robotic platform for operation. Notice that this BCI aims to recognize when the individual passively walking in Lokomat is performing gait motor imagery, and teach him/her the modulation of both *µ* and *β* rhythms over the motor cortex, considering user-specific cortical activity patterns, as shown in our previous published report by Blanco-Diaz et al., (2024)^38^. On the other hand, Donati et al. (2016)^40^ and Shokur et al. (2018)^42^conducted active gait training in SCI individuals using two main brain-machine interfaces (BMIs) paradigms with actuators as Lokomat and a custom-made robotic exoskeleton. In this proposal, MI was performed entirely at rest and classification is binary (left or right leg movement), providing only a right-or-wrong indication of the user’s cortical state. Among other results, Shokur et al. (2018)^42^ reported reduction of NB symptoms after individuals command left and right legs MI-based BMI. Our findings corroborate with the results of this study, but adding the following scientific contributions (1) Riemannian covariance features are used to accurately discriminate both walking without MI and walking with MI; (2) refinement of the mathematical formulation, specifically considering both *µ* and *β* rhythm oscillations, is proposed to cancel the default cortical effect that lokomat generates when providing passive gait training. In our study this formulation is applied using firstly a single-channel NFB, and immediately later the same proposed formulation is used to obtain a multi-channel NFB model; (3) ability to support continuous BCI re-calibration adaptation, allowing the system to recalibration is proposed after each trial to adjust the multi-channel EEG neurofeedback model. We believe that continuous NFB on cortical activity provided by our BCI system, as opposed to binary feedback, may be more effective in promoting the user’s cognitive engagement during MI walking, leading to more precise cortical activation. Thus, the improvement in the NBSS results can be attributed to the enhanced cortical activation, observed after a short intervention period in our research, compared to previous BMI.

It is important to note that our protocol used 100% guidance force on the Lokomat, and the BCI system was calibrated to recognize the MI of walking and associate the relative power changes of the EEG, enhancing the training’s accuracy. To date, we believe that the protocol used is a pioneer in improving the modulation of *µ* and *β* rhythms alongside clinically meaningful gains in bladder function, assessed by NBSS, in individuals with motor-complete SCI.

In the present study, we observed correlations between cortical modulation of *µ* and *β* rhythms and NBSS domains further support the hypothesis that cortical engagement through MI-NFB training contributed to the clinical gains. There was a strong negative correlation between EEG locations in the frontal lobe and improvement in NBSS scores, specifically in the premotor and supplementary motor cortex areas associated with increased *µ* and *β* relative power changes. It has been shown that the induction of a micturition attempt elucidates an activation pattern in key brain regions, which are crucial in processing visceral sensations and regulating emotional and autonomic responses^44^.

In this context, the frontal lobe area plays a fundamental role in the micturition process^20^. Specifically, the prefrontal cortex is important for directing attention to vesical sensations and making decisions related to micturition^44^. The prefrontal relates to other brain and brainstem areas involved in the conscious control of bladder function^17^. Further, the primary motor cortex has long been recognized as a hub for voluntary movement control, and accumulating evidence suggests its broader involvement in diverse motor behaviors, including both locomotion and urination. Yao et al. (2018)^45^ identified a corticopontine circuit originating from the primary motor cortex that is crucial for the initiation of urination via direct projections to the pontine micturition center. In an electrophysiological study, spinal cord stimulation applied to healthy rats modulated cortical rhythms in primary motor cortex, particularly reducing *α* and *β* rhythms during gait initiation^46^. These oscillatory changes are consistent with a facilitative cortical state for initiating voluntary movement and strengthens the modulatory role of primary motor cortex in bladder voiding described by Yao et al. (2018)^45^. This suggests a shared neurophysiological mechanism through which the primary motor cortex engages and disinhibits downstream motor circuits.

Clinically, this integrative role of primary motor cortex has been explored by Qian et al.^47^who reported that repetitive transcranial magnetic stimulation over primary motor cortex improved bladder function in a post-stroke patient. Together, these findings propose that the primary motor cortex may act as a convergent node that governs multiple motor outputs, potentially through selective top-down control of both spinal and brainstem targets.

The parallel modulation of locomotor and urinary functions via primary motor cortex further underscores the therapeutic potential of cortical neuromodulation, whether through transcranial or spinal stimulation, in managing complex motor disorders. This convergence opens new perspectives for integrative rehabilitation protocols targeting the primary motor cortex to simultaneously improve gait and urinary outcomes in patients with neurological impairments. Therefore, it is possible to suggest that modulation of *µ* and *β* rhythms led to an improvement in NB symptoms in individuals with complete SCI. Moreover, the findings of the present study are particularly relevant when considering previous work that also demonstrated cortical modulation and clinical improvements using Lokomat and NFB^40^in special because changes in *µ* suggests an improvement in neuronal communication and sensorimotor integration, fundamental to motor control and sensory perception^48,49^.

Interestingly, brain oscillations were more expressive at the midpoint of the intervention, while the beneficial clinical outcomes persisted for a long time. It is possible to suggest an optimized therapeutic response potentially related to a period of increased neuronal plasticity following injury. Such findings are particularly relevant for future rehabilitation programs, as they indicate that the intensity of interventions may play a crucial role in maximizing therapeutic benefits^50^possibly due to enhanced neuronal plasticity^49^. In this context, the results provide novel evidence of cortical reorganization induced by combined therapies with neurofeedback in individuals with SCI.

The intervention did not cause an improvement in the ASIA (trunk sensorial scores) and the beneficial results obtained for NBSS did not correlate with trunk sensibility and balance. Although the protocol induced beneficial results in the mFRT, no correlation was observed with the NBSS scores. Undoubtedly, our study contributes to the current understanding of potential therapeutic pathways.

A limitation of this study is the absence of a control group, which prevents us from fully ruling out the hypothesis of spontaneous recovery. However, we provide several reasons to suggest that spontaneous recovery does not account for the observed effects. All participants had chronic motor-complete SCI, whose probability of natural neurological recovery (i.e., ASIA A to B or to C) is significantly more restricted^51^. Furthermore, considering that preserved sensation in the S3–S5 and T11–L2 dermatomes is associated with awareness of bladder filling and the potential for voluntary voiding^52^the absence of these sensory predictors prior to intervention reinforces the notion of limited natural recovery potential in the seven participants included in the study. Finally, in the users’ previous clinical history, there were no reports of changes in NBSS up to the start of the present study and participants were not receiving any concurrent pharmacological or neuromodulatory treatments during our intervention. Therefore, the consistent and clinically meaningful improvements observed, especially the MCID being reached in six out of seven individuals are unlikely to reflect natural recovery, and instead point to a therapeutic effect of the MI-BCI combined with robotic-assisted gait training.

## Conclusion

The present study showed that improving the *µ* and *β* cortical modulation using robotic gait training with a MI based BCI leads to improvement of NBSS symptoms in SCI individuals.

## Limitations

This study has some limitations, including a small sample size, the absence of a control group, and the lack of urodynamics post-intervention, which is the gold standard for evaluating the NB.

## Supplementary Information

Below is the link to the electronic supplementary material.

Supplementary Material 1

## Data Availability

The datasets used and/or analyzed during the current study are available from the corresponding author on reasonable request.

## Abbreviations

ANS: Autonomic nervous system
ASIA: American spinal injury association
BCI: Brain-computer interface
CAAE: Certificado de Apresentação para Apreciação Ética
CAR: Common average reference
CNS: Central nervous system
EEG: Electroencephalography
KVIQ-10: Kinesthetic and visual imagery questionnaire-10
MAS: Modified ashworth scale
mFRT: Modified functional reach test
MI: Motor imagery
MCID: Minimal clinically important difference
NBSS: Neurogenic bladder symptom score
NFB: Neurofeedback
NLUTD: Neurogenic lower urinary tract dysfunction
PAG: Periaqueductal gray area
PFM: Pelvic floor muscles
PNS: Peripheral nervous system
PSD: Power spectral density
SCI: Spinal cord injury
SEM: Standard error of the mean
SMR: Sensorimotor rhythm
