# Supplementary material for: Cortical modulation through robotic gait training with motor imagery brain-computer interface enhances bladder function in individuals with spinal cord injury
Source: Sci Rep. 2025 Oct 3;15:34633. doi: 10.1038/s41598-025-18277-3 (PMC12494983; doi:10.1038/s41598-025-18277-3)
Supplement: Supplementary file 1 — Supplementary Material 1 [file 41598_2025_18277_MOESM1_ESM.docx]

Supplementary materials

Table 1. Trunk sensorial scores.

| **Subjects** | **Pre-therapy** | **Midpoint** | **Post-therapy** | **Follow-up** |
| --- | --- | --- | --- | --- |
| Subject 1 | 77 | 78 | 78 | 77 |
| Subject 2 | 57 | 56 | 66 | 63 |
| Subject 3 | 87 | 88 | 92 | 90 |
| Subject 4 | 65 | 63 | 61 | 64 |
| Subject 5 | 82 | 82 | 87 | 86 |
| Subject 6 | 42 | 55 | 46 | 46 |
| Subject 7 | 47 | 64 | 81 | 81 |

Table 2. Values of mFRT.

| **Subjects** | **Evaluations** | **Front** | **Right side** | **Left side** |
| --- | --- | --- | --- | --- |
| **Subject 1** | Pre-therapy | 0 | 17 | 13 |
|  | Midpoint | 2 | 17 | 15 |
|  | Post-therapy | 2 | 20 | 19 |
|  | Follow-up | 2 | 17 | 19 |
| **Subject 2** | Pre-therapy | 0 | 6 | 3 |
|  | Midpoint | 0 | 7 | 4 |
|  | Post-therapy | 2 | 7 | 6 |
|  | Follow-up | 2 | 6 | 5 |
| **Subject 3** | Pre-therapy | 15 | 19 | 12 |
|  | Midpoint | 13 | 20 | 13 |
|  | Post-therapy | 20 | 23 | 18 |
|  | Follow-up | 18 | 20 | 17 |
| **Subject 4** | Pre-therapy | 1 | 7 | 11 |
|  | Midpoint | 1 | 5 | 8 |
|  | Post-therapy | 5 | 6 | 12 |
|  | Follow-up | 5 | 4 | 10 |
| **Subject 5** | Pre-therapy | 0 | 0 | 0 |
|  | Midpoint | 0 | 0 | 0 |
|  | Post-therapy | 0 | 0 | 0 |
|  | Follow-up | 0 | 0 | 0 |
| **Subject 6** | Pre-therapy | 5 | 18 | 14 |
|  | Midpoint | 6 | 19 | 18 |
|  | Post-therapy | 14 | 25 | 24 |
|  | Follow-up | 13 | 25 | 20 |
| **Subject 7** | Pre-therapy | 3 | 5 | 12 |
|  | Midpoint | 4 | 6 | 14 |
|  | Post-therapy | 7 | 9 | 16 |
|  | Follow-up | 6 | 6 | 13 |
